# Effects of a low FODMAP diet on the colonic microbiome in irritable bowel syndrome: a systematic review with meta-analysis

**DOI:** 10.1093/ajcn/nqac176

**Published:** 2022-06-21

**Authors:** Daniel So, Amy Loughman, Heidi M Staudacher

**Affiliations:** Department of Gastroenterology, Central Clinical School, Monash University and Alfred Health, Melbourne, Victoria, Australia; Food & Mood Centre, Institute for Mental and Physical Health and Clinical Translation (IMPACT), Deakin University, Geelong, Victoria, Australia; Food & Mood Centre, Institute for Mental and Physical Health and Clinical Translation (IMPACT), Deakin University, Geelong, Victoria, Australia

**Keywords:** FODMAP, irritable bowel syndrome, colonic microbiota, colonic microbiome, short-chain fatty acids

## Abstract

**Background:**

A low fermentable oligosaccharides, disaccharides, monosaccharides, and polyols (FODMAP) diet is increasingly used to manage symptoms in irritable bowel syndrome (IBS). Although this approach may alter the colonic microbiome, the nature of these changes has not been comprehensively synthesized.

**Objectives:**

The aim of this study was to conduct a systematic review with meta-analysis of randomized controlled trials examining the impact of a low FODMAP diet on the composition and function of the microbiome in patients with IBS.

**Methods:**

A systematic search was conducted for randomized controlled trials evaluating the effects of a low FODMAP diet on the colonic microbiome in patients with IBS in MEDLINE, EMBASE, CENTRAL, and Web of Science from inception to April 2022. Outcomes included diversity of the microbiome, specific bacterial abundances, fecal SCFA concentration, and fecal pH. For fecal SCFA concentrations and pH, meta-analyses were performed via a random-effects model.

**Results:**

Nine trials involving 403 patients were included. There were no clear effects of the low FODMAP diet on diversity of the microbiome. A low FODMAP diet consistently led to lower abundance of Bifidobacteria, but there were no clear effects on diversity of the microbiome or abundances of other specific taxa. There were no differences in total fecal SCFA concentration between the low FODMAP diet and control diets (standardized mean difference: −0.25; 95% CI: −0.63, 0.13; *P* = 0.20), nor were there differences for fecal concentrations of specific SCFAs or fecal pH.

**Conclusions:**

In patients with IBS, the effects of a low FODMAP diet on the colonic microbiome appear to be specific to Bifidobacteria with no consistent impacts on other microbiome metrics, including diversity, fecal SCFA concentrations, and fecal pH. Further, adequately powered trials are needed to confirm these findings.

This review was registered at https://www.crd.york.ac.uk/prospero/ as CRD42020192243.

## Introduction

Irritable bowel syndrome (IBS) is a common disorder of gut–brain interaction associated with substantial compromise to quality of life (1) and incurs considerable economic burden (2). The pathophysiology of IBS is incompletely understood but multiple factors including visceral hypersensitivity, alterations to the gastrointestinal microbiome, and dysfunction of the gut–brain axis are postulated to be key features (3).

Dietary approaches for the management of symptoms in IBS are of specific interest because most patients consider their symptoms to be related to food (4, 5). Of these approaches, multiple syntheses have shown that a diet low in fermentable oligosaccharides, disaccharides, monosaccharides, and polyols (FODMAPs) is efficacious for reducing gastrointestinal symptoms in this patient group (6–8).

Shifts to the microbiome have been documented in response to a low FODMAP diet, which has led to questions about the safety of the diet (9, 10). Changes reported previously include reduction in putatively beneficial *Bifidobacteria* in patients with IBS (8) as well as shifts in the overall microbiome composition toward dysfunction in patients with gastrointestinal diseases (11). Furthermore, a low FODMAP diet may also lead to deleterious alterations in microbial metabolism, reflected by reduced concentrations of fecal SCFAs (12), although whether such effects occur consistently across all trials is unknown.

Such shifts to the composition and metabolism of the microbiome are of particular relevance given the microbiome aberrations documented in IBS (13). A comprehensive synthesis of these shifts is required to clarify the extent to which a low FODMAP diet affects the microbiome, in order to better inform on safety and help elucidate potential mechanisms of its therapeutic effect.

## Methods

### Literature search

This systematic review was conducted according to a prospectively registered protocol (CRD42020192243). Systematic searches were performed in MEDLINE, EMBASE, CENTRAL, and Web of Science (to 18 April, 2022) (**Supplemental Tables 1–4**). Results were merged into the Covidence software (Veritas Health Innovation) and de-duplicated, with abstract screening and full-text review for eligibility conducted independently by 2 authors (DS, HMS). Disagreements in judgment were resolved by a third reviewer (AL).

### Study selection

Trials were included if they *1*) were a randomized controlled trial; *2*) included adult patients (≥18 y of age) with a diagnosis of IBS; *3*) evaluated a low FODMAP diet; *4*) included a control diet; *5*) lasted ≥7 d; *6*) and evaluated 1 of the following outcomes after intervention: global composition of the microbiome, bacterial abundances, mycobiome and virome composition, fecal SCFA concentration, fecal pH, or breath gas concentration. Attempts were made to contact the corresponding author when the full-text article provided inadequate information to allow the extraction of relevant data. Trials that included multiple patient groups, where findings for the IBS subgroup were not reported separately, were excluded.

The primary outcome was between-group differences in the global composition of the microbiome, evaluated using α-diversity or β-diversity metrics, after intervention. Secondary outcomes were within-group comparisons of the global composition of the microbiome, between baseline and after intervention, as well as between- and within-group comparisons of the following: mycobiome and virome metrics, bacterial load (total bacterial count), specific bacterial abundances, fecal SCFAs [total and individual, including branched-chain fatty acids (BCFAs)], fecal pH, and breath gas concentration. For bacterial abundances, only comparisons for taxa in which findings were reported by ≥2 trials were extracted.

### Data extraction and management

Two reviewers (DS, HMS) independently extracted the data from eligible studies. Data extracted included details of study design, patient characteristics, details of intervention, and controls. For prespecified outcomes, the mean and variance reported as end of intervention values were extracted for analysis. Risk of bias was independently assessed by 3 reviewers (AL, DS, and HMS) using Cochrane methodology (14).

### Statistical analysis

The overall treatment effect of interventions on outcomes was calculated using the difference between end of intervention values for the intervention compared with control groups. Where these data were unable to be obtained or were not suitable for meta-analysis, results were narrated.

Meta-analysis was performed when outcomes were reported in ≥2 trials with Revman version 5.3 (Cochrane Collaboration). The mean difference (MD) was used to calculate effect sizes where outcome data were presented using the same units and standardized mean difference (SMD) where outcome data were reported in different units. A random-effects model was used to produce a pooled estimate of the MD or SMD. Heterogeneity between studies was assessed using the *I*^2^ statistic, with significant heterogeneity defined as *I*^2^ ≥50%.

## Results

### Study characteristics

**FIGURE 1:**
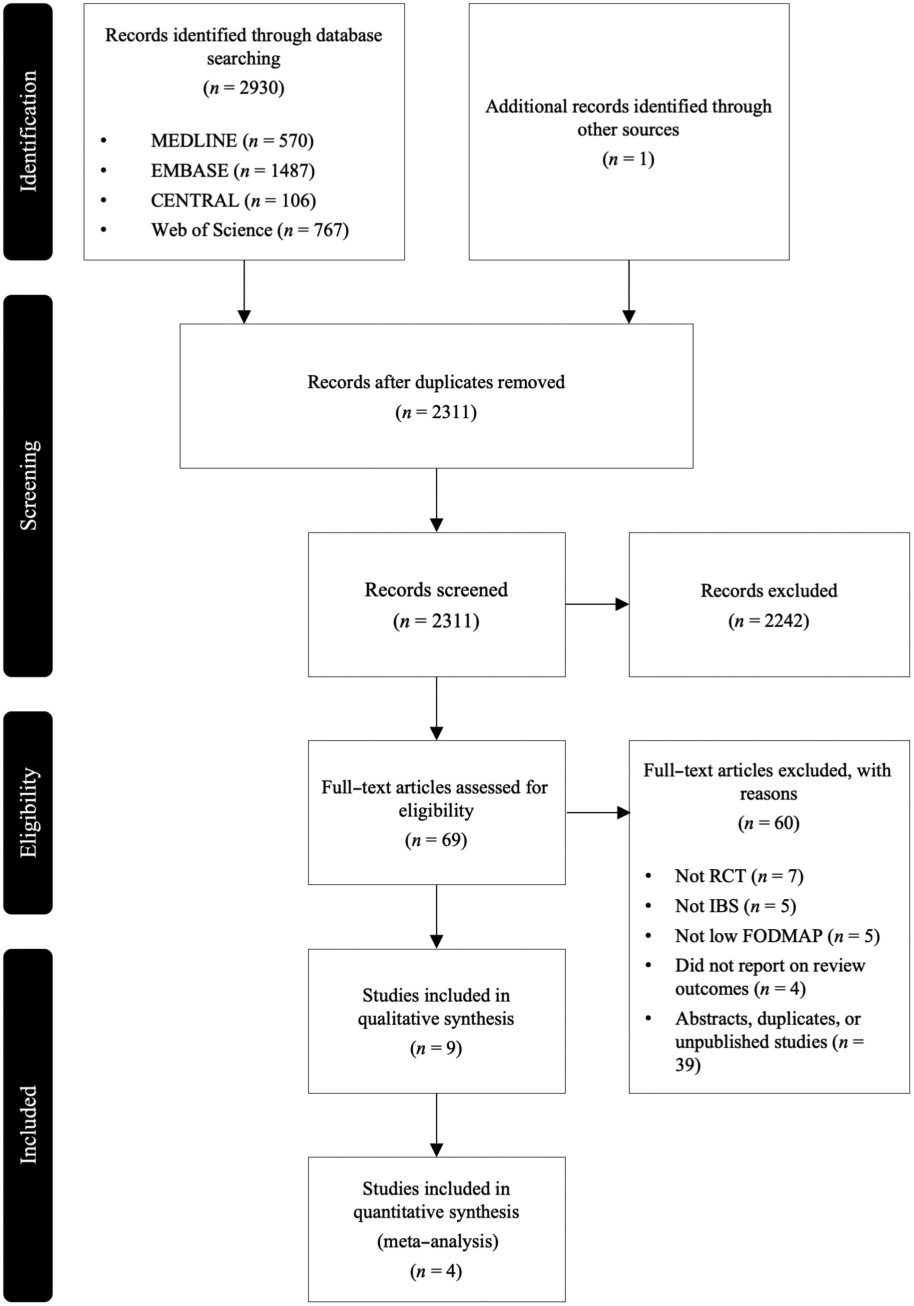
Flow diagram of studies evaluated in the systematic review. FODMAP, fermentable oligosaccharides, disaccharides, monosaccharides, and polyols; IBS, irritable bowel syndrome; RCT, randomized controlled trial.

The systematic searches identified 2930 publications (**Figure 1**). After full-text review, 9 trials (12, 15–22) and 1 secondary publication (23) were included. Additional data were obtained from investigators of 2 trials (16, 20). All 9 trials reported on composition of the microbiome, 5 reported on concentrations of SCFAs and BCFAs (12, 16, 19–21), and 4 reported on fecal pH (12, 16, 19, 20). No trials reported on mycobiome, virome, or breath gas concentration.

A total of 403 patients were analyzed across trials conducted in the United Kingdom (12, 19, 20, 22), Australia (16), Canada (15), China (21), New Zealand (17), and Sweden (18). Most trials used Rome III IBS diagnostic criteria and one used Rome IV criteria (22). One trial included only patients with diarrhea-predominant IBS (21), 3 trials excluded patients with constipation-predominant IBS (12, 19, 20), and 1 trial included patients with diarrhea-predominant and mixed-type IBS (22). Eight trials delivered the low FODMAP intervention via dietary advice from dietitians using a parallel design (12, 15, 17–22), and 1 trial used controlled feeding in a crossover design, where most food was provided to patients (**Table 1**) (16). Five trials were single-blind (12, 15, 16, 18, 20) and 4 trials were unblinded (17, 19, 21, 22). Most trials ran for 3–4 wk (12, 15, 16, 18–22) and 1 trial lasted for 3 mo (17).

**TABLE 1:** Characteristics of randomized controlled trials of low FODMAP diet intervention in patients with irritable bowel syndrome^1^

		Patients		Interventions			Trial design				
Trial (reference)	Trial location	*n*; age^2^; % M	Diagnosis	Intervention diet delivery	Control diet; diet delivery	Adherence	Design	Duration, d	Blinding	Washout, d	Microbiome analysis methods
Bennet et al. (15)	Sweden	61; 18–69; 19	Rome III	Dietary advice	NICE/BDA guidelines; dietary advice	Unclear	Parallel	28	Single	N/A	GA-map Dysbiosis Test
Halmos et al. (16)	Australia	27; 41; 22	Rome III	Controlled feeding	Typical Australian diet; controlled feeding	80%	Crossover	21	Single	21	DGGE; qPCR
Harvie et al. (17)	New Zealand	50; 42; 14	Rome III	Dietary advice	Nil (waitlist)	Not assessed	Parallel	90	Unblinded	N/A	16S rRNA sequencing
McIntosh et al. (18)	Canada	27; 24–83; 14	Rome III	Dietary advice	High FODMAP diet; dietary advice	Unclear	Parallel	21	Single	N/A	16S rRNA sequencing
Rej et al. (22)	United Kingdom	35; 38; 26	Rome IV (IBS-D, IBS-M)	Dietary advice	NICE/BDA guidelines; dietary advice	Unclear	Parallel	28	Unblinded	N/A	GA-Map Dysbiosis Test
Staudacher et al. (19)	United Kingdom	35; 35; 37	Rome III (no IBS-C)	Dietary advice	Habitual diet; dietary advice	Unclear	Parallel	28	Unblinded	N/A	FISH
Staudacher et al. (20, 23)	United Kingdom	40; 34; 31	Rome III (no IBS-C)	Dietary advice	Sham diet; dietary advice	100%	Parallel	28	Single	N/A	16S rRNA sequencing; qPCR
Wilson et al. (12)	United Kingdom	42; 35; 44	Rome III (no IBS-C)	Dietary advice	Sham diet; dietary advice	91%–95%	Parallel	28	Single	N/A	16S rRNA sequencing; FISH
Zhang et al. (21)	China	86; 44; 53	Rome III (IBS-D only)	Dietary advice	NICE/BDA guidelines; dietary advice	78%	Parallel	21	Unblinded	N/A	16S rRNA sequencing

1 BDA, British Dietetic Association; DGGE, denaturing gradient gel electrophoresis; FISH, fluorescence in situ hybridization; FODMAP, fermentable oligosaccharides, disaccharides, polysaccharides, and polyols; GA, Genetic Analysis; IBS-C, constipation-predominant irritable bowel syndrome; IBS-D, diarrhea-predominant irritable bowel syndrome; IBS-M, mixed-type irritable bowel syndrome; N/A, not applicable; NICE, National Institute for Health and Care Excellence; rRNA, ribosomal RNA.

2 Age expressed as mean years; age range provided where means were not reported.

All trials evaluated the fecal microbiome using a range of techniques: 16S ribosomal RNA (rRNA) sequencing (17, 18, 21); fluorescence in situ hybridization (FISH) (19); and the Genetic Analysis-map Dysbiosis Test, a qPCR technique (15, 22). Three trials used a combination of techniques: denaturing gradient gel electrophoresis with qPCR (16); 16S rRNA sequencing with qPCR (20, 23); and 16S rRNA sequencing with FISH (12). Fecal SCFA and BCFA concentrations were assessed via GC (16, 21) and GLC (12, 19, 20), and fecal pH via calibrated probes (12, 16, 19, 20).

**FIGURE 2:**
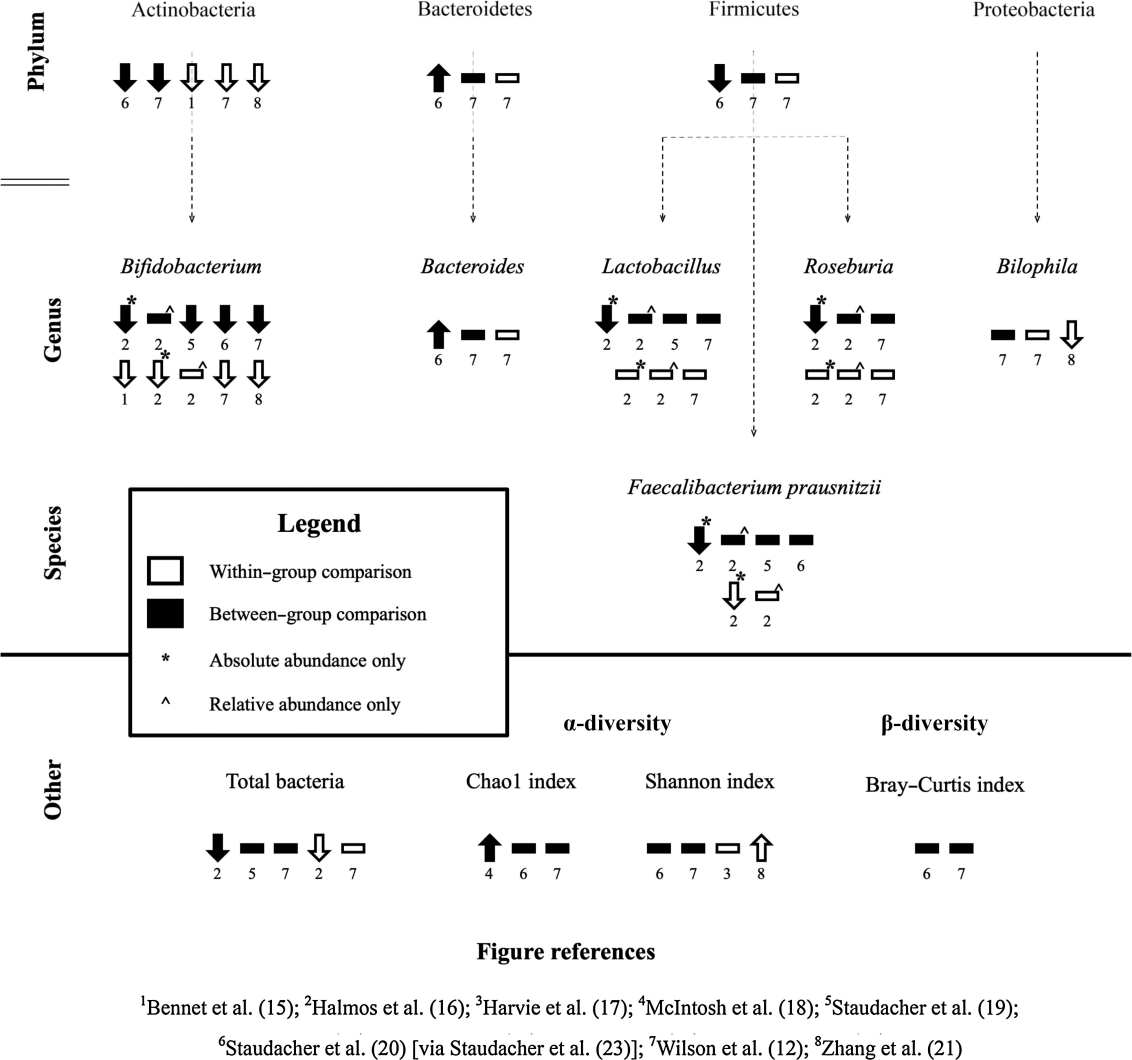
Summary of microbiome outcomes of 8 trials included in the review, excluding data for Rej et al. (22). The trials evaluated taxonomy using either absolute or relative abundance, with the exception of Halmos et al. (16), which reported both metrics separately and the results are presented accordingly, with symbols used to denote absolute (*) and relative (^) abundance data (see Legend).

Microbiome data reported by included trials were not statistically pooled owing to the heterogeneity of analysis techniques, and the need to subject raw data to the same bioinformatic preprocessing procedures for valid comparisons (24). Meta-analysis of trials with such heterogeneous microbiome methodology from collection, sequencing, preprocessing, filtering, and reporting would not provide useful estimates of effect size given the large contribution of methodology to the results. Even estimates of α-diversity are difficult to validly compare between studies, because they are subject to bias by sampling depth, which is inconsistently reported and rarely adjusted for in analyses (25). Findings were instead summarized in **Figure 2** and narrated. In 1 trial, bacterial abundances were reported in comparison to a “normobiotic reference range” (22). These data were not summarized in Figure 2 because this qualitative descriptor could not be meaningfully compared to analysis methods used in other studies, and were narrated if the taxon was reported on by ≥2 other included trials. Meta-analysis was performed for fecal SCFA and BCFA concentrations and fecal pH.

### Global microbiome composition (α-diversity and β-diversity)

α-Diversity of the microbiome was assessed in 5 trials (12, 17, 18, 21, 23) and β-diversity in 3 trials (12, 20, 21).

Three dietary advice trials reported between-group comparisons of α-diversity using the Chao1 index. In 1 trial, α-diversity was higher after intervention in the low FODMAP diet group than in the high FODMAP diet control group (18), whereas the other trials found no difference in comparison with sham dietary advice groups (12, 23). The latter 2 trials also reported no between-group differences in α-diversity using the Shannon index (12, 23). Two dietary advice trials reported within-group comparison of α-diversity using the Shannon index. One trial found no within-group change (17), whereas another reported α-diversity to be higher after 3 wk of intervention than at baseline (21).

Two trials reported within-group comparisons in β-diversity using the Bray–Curtis dissimilarity index. Both reported no change at 4 wk (12, 20). One trial found no within-group change in β-diversity using principal coordinates analysis (21).

### Bacterial load

The impact of a low FODMAP diet on bacterial load was evaluated by 3 trials (12, 16, 19). Two dietary advice trials evaluated between-group comparisons, reporting no difference in bacterial load after a low FODMAP diet compared with habitual diet (19) or sham dietary advice (12). One feeding trial and 1 dietary advice trial reported a within-group comparison for bacterial load. In the feeding trial, bacterial load was lower than at baseline (16), but there was no difference in the dietary advice trial (12).

### Abundances of phylum-level taxa

The effects a low FODMAP diet on bacterial abundance at the phylum level were reported by 5 dietary advice trials (12, 15, 20–22). Two trials reported between-group comparisons of phyla abundance for low FODMAP dietary advice and sham dietary advice, with a lower Actinobacteria abundance in the low FODMAP groups after intervention in both trials (12, 23), but the findings for other phyla were inconsistent. One trial reported a higher abundance of Bacteroides and lower abundance of Firmicutes in the low FODMAP group than in the sham (23), whereas no between-group differences were found for these phyla in the other trial (12).

Within-group comparisons for bacterial abundance at the phylum level were reported in 3 trials. All trials reported lower Actinobacteria abundance after intervention than at baseline (12, 15, 21), whereas no differences for Bacteroides or Firmicutes were found (12).

In the dietary advice trial that reported within-group comparisons based on abundance relative to a “normobiotic reference range,” phylum-level comparisons showed Actinobacteria to be lower after intervention with respect to the reference range, whereas there was no difference for Firmicutes (22).

### Abundances of genus-level taxa

At the genus level, the most commonly reported taxa across all included trials were *Bifidobacteria* and *Lactobacillus*. Seven trials reported on the abundance of *Bifidobacteria*. In the trials reporting between-group comparisons for *Bifidobacteria*, there was a lower abundance in the low FODMAP diet group after intervention than in the control diet groups (12, 16, 19, 20). Four trials reported within-group comparisons for *Bifidobacteria*, all reporting a lower abundance of *Bifidobacteria* after a low FODMAP diet than at baseline (12, 15, 16, 21).

Four trials reported the effect of the low FODMAP diet on abundance of *Lactobacillus*. In 2 trials there was no between-group difference in *Lactobacillus* abundance between the low FODMAP diet groups and control diet groups (12, 19). Two trials reported no within-group change in Lactobacillus abundance after a low FODMAP diet compared with baseline (12, 16).

The effects of a low FODMAP diet on other genera were less commonly reported. Two trials reported no between-group differences in *Roseburia* spp. (12, 16), although 1 found absolute but not relative abundance to be lower than with the control diet (16). Two dietary advice trials reported on abundances of *Bacteroides* spp. with inconsistent findings. *Bacteroides* abundance was higher in the low FODMAP group than in the sham group after intervention in 1 trial (23) but no difference was reported in the other sham-controlled trial (12). Two dietary advice trials reported within-group comparisons of *Bilophila* spp. One reported higher abundance than at baseline (21), whereas no difference was found in the other (12).

In the dietary advice trial that reported within-group comparisons based on abundance relative to a “normobiotic reference range,” genus-level comparisons showed no differences for *Bifidobacteria, Lactobacillus*, or *Bacteroides* spp. after intervention (22).

### Abundances of species-level taxa

The effects of interventions on *Faecalibacterium prausnitzii* were reported by 4 trials (16, 19, 22, 23). Two dietary advice trials found no between-group difference after intervention in this bacterial species (19, 23). In the feeding trial, there was no difference in *F. prausnitzii* abundance after intervention compared with baseline (16). One dietary advice trial reported no difference in *F. Prausnitzii* after intervention compared with baseline relative to the “normobiotic reference range” (22).

### Fecal metabolites

Five trials evaluated the effect of a low FODMAP diet on fecal SCFA and BCFA concentrations, 4 of which were suitable for meta-analysis (12, 16, 19, 20). There was no difference in the concentration of total or individual SCFAs or BCFAs between low FODMAP and control diets after intervention, with moderate heterogeneity observed (**Table 2**). Within-group comparisons were reported in the trial not included in meta-analysis (21), in which butyrate concentration was lower and iso-butyrate and iso-valerate were higher at the end of a 3-wk intervention than at baseline, and acetate and propionate did not change (21).

**TABLE 2:** Total fecal SCFA, individual SCFA and fecal pH reported in ≥2 randomized controlled trials and included in the meta-analysis^1^

	Results		Heterogeneity		
Outcomes	Meta-analysis overall estimate (95% CI)	*P*	Chi-square test	*P*	*I* ^2^, %
Total fecal SCFAs	SMD: −0.25 (−0.63, 0.13)	0.20	5.91	0.12	49
Acetate	SMD: −0.24 (−0.60, 0.12)	0.18	5.36	0.15	44
Propionate	SMD: −0.18 (−0.56, 0.20)	0.35	5.94	0.11	49
Butyrate	SMD: −0.30 (−0.68, 0.08)	0.13	5.99	0.11	50
Valerate	SMD: −0.22 (−0.53, 0.10)	0.18	4.15	0.25	28
Iso-butyrate	SMD: 0.02 (−0.23, 0.28)	0.86	0.47	0.93	0
Iso-valerate	SMD: 0.00 (−0.26, 0.25)	0.98	1.31	0.73	0
Fecal pH	MD: 0.26 (−0.08, 0.60)	0.14	4.87	0.18	38

1 Data were meta-analyzed using a random-effects model and presented as MD or SMD as appropriate. Statistical heterogeneity was assessed using the chi-square test and quantified using the *I*^2^ statistic. All meta-analyses were informed by 4 trials involving 208 participants (12, 16, 19, 20). MD, mean difference; SCFA, short-chain fatty acids; SMD, standardized mean difference.

Fecal pH was assessed in 4 trials. All trials reported no differences in pH after intervention compared with control diets (Table 2) (12, 16, 19, 20).

### Risk of bias

**FIGURE 3:**
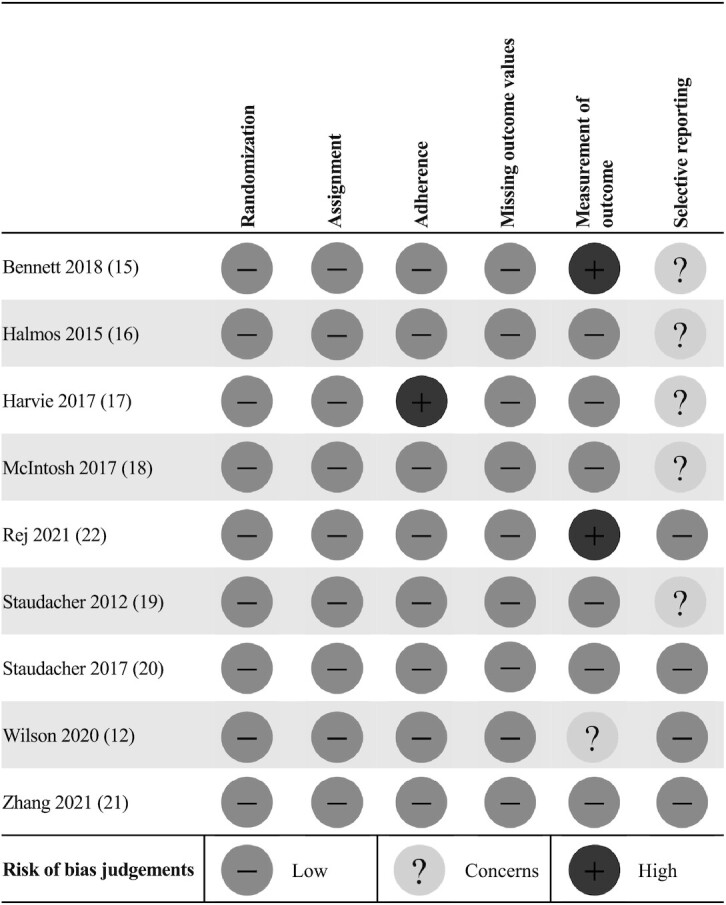
Summary of risk of bias judgments across the included trials according to the Cochrane Risk of bias 2.0 tool.

Risk of bias across the included trials was generally low (**Figure 3**). The risk of bias arising from randomization and assignment were low. Concerns with bias related to adherence to interventions were identified in 1 trial where adherence data were not reported (17). The risk of bias arising from missing outcome data was low. Potential bias arising from measurement of outcomes was low in all but 3 trials, where no information was reported about the outcome assessor blinding for subjective assessments of bacterial abundances [bacterial counts (15, 22) and fluorescent signal detection (12)]. There were concerns for potential bias resulting from selective reporting in most trials. Only 4 trials prospectively registered in a clinical trial registry describing a priori planned outcomes (12, 20–22).

## Discussion

This is the most comprehensive systematic review to date reporting the effect of a low FODMAP diet on colonic microbiome composition and function in IBS. This is important for informing on safety, and holds potential importance for understanding the mechanisms underlying symptom response in this patient group. Aside from a clear reduction in *Bifidobacteria*, the dietary approach led to inconsistent or minimal effects on microbiome composition and metabolism.

Overall, the α-diversity of the microbiome was not affected by a low FODMAP diet. These findings are reassuring considering higher diversity has been considered a hallmark of gastrointestinal health and lower diversity associated with disease states (26). Moreover, β-diversity was also not altered by the dietary approach, suggesting the overall composition of the microbiome may not be appreciably altered by low FODMAP dietary intervention.

Similarly, a low FODMAP diet did not exert clear effects on bacterial load. This finding is notable, given the premise of this dietary approach involves reducing the availability of fermentable carbohydrates in the colon (27), limiting the major substrates available for microbial proliferation (28). This result suggests that FODMAPs may be preferentially metabolized by specific bacteria, such as *Bifidobacteria*, and potentially taxa not measured in these trials, rather than by the majority of commensal organisms. FODMAP restriction therefore leads to a relatively stable number of total organisms, and indeed overall diversity of the community, despite a reduction in total carbohydrate substrates entering the colon.

The most striking finding was the effect of a low FODMAP diet on *Bifidobacteria* and its phylum Actinobacteria, with abundances consistently lower after intervention than after control diets and/or at baseline. The metabolic repertoire of *Bifidobacteria*, which includes the ability to degrade a wide range of fibers, including fructans, may explain these effects (29). As part of a low FODMAP diet, consumption of these short-chain fibers is specifically restricted, whereas fructan supplementation, at least in healthy individuals, appears to selectively stimulate growth of *Bifidobacteria* (30).

This “antibifidogenic” effect of the low FODMAP diet has been an area of concern. *Bifidobacteria* have putative immunomodulatory and anticancer properties in animal studies (31, 32), with antitumor effects via enhanced T-cell activation shown in mice (32). In humans, a lower abundance has been associated with greater symptom severity in IBS (33). Attempts have been made to prevent these alterations in the short term. Concomitant supplementation with a *Bifidobacteria*-containing probiotic helped ameliorate this effect (20) but low-dosage fiber supplementation (1.4 g/d β-galacto-oligosaccharide) did not (12). Importantly, the included trials only examined short-term FODMAP restriction. The low FODMAP diet is intended to be delivered as a short-term intervention followed by reintroduction of restricted FODMAPs and personalization (34). Whether this antibifidogenic effect persists in the long term is critical when considering safety. One recent small follow-up trial reported restoration of *Bifidobacteria* abundance after the personalization phase (35); however, larger studies are required to confirm this finding.

The lack of effect of a low FODMAP diet on fecal SCFA concentrations and pH could be interpreted as an extension of the lack of wide-ranging effects on microbiome composition. However, accurate assessment of SCFAs and pH is a challenge, because fecal concentration is more reflective of the rectal environment rather than the colon overall. Furthermore, the fecal concentration may not even be reflective of the luminal concentration, given the majority of carbohydrate fermentation and therefore SCFA production occurs in the proximal colon (27), and that SCFAs are generally absorbed at the site of production (36). Direct measures of microbiome metabolism (e.g., telemetric capsules) (37, 38), would considerably advance understanding of the effects of diet on microbiome function.

The risk of bias of trials was generally judged to be low. This is in contrast with previous systematic reviews of the same trials evaluating symptom endpoints, where bias concerns relating to the blinding of patients and outcome assessors (7, 39) and choice of control diets (39) have been raised. The reasons for such disparity are 2-fold. Firstly, this review focused on microbiome outcomes that are generally assessed objectively, whereas symptom outcomes are assessed subjectively and are more prone to biases related to lack of blinding (40). Secondly, previous bias assessments have attempted to apply metrics for pharmaceutical research to nutrition trials (7, 39), which fails to acknowledge nuances of nutrition research, such as the challenge of blinding whole-diet interventions and incorporating appropriate controls (40–42), and thus may not be appropriate for appraisal of lifestyle interventions in IBS (43). Although a similar degree of rigor was applied in this review, the impact of blinding was less contentious as already discussed, and additional risk of bias criteria were not specifically introduced to critique the choice of control groups, as previously applied (39).

This study is, to date, the most comprehensive synthesis of trials reporting microbiome responses to a low FODMAP diet in IBS. A major strength of this review is the use of multiple microbiome endpoints, because the use of specific isolated metrics may not convey the breadth of effects on the microbial community (44). Further, the effect on microbial function was also included because it is clear that metabolism of the microbiome in addition to taxonomy is important for understanding the consequences for health (44).

There are some limitations to consider. Firstly, only a small number of trials were included. The reported outcomes varied and, aside from abundances of *Bifidobacteria* and *Lactobacillus*, other microbiome metrics were only reported in a small proportion of trials. Secondly, there was substantial heterogeneity in trial design. For example, there was variability in the mode of delivery and control diets used. Furthermore, owing to the range of techniques used to assess the microbiome, taxonomic data were reported in absolute and relative abundance across trials, as well as abundance relative to a reference range derived from a predominantly Scandinavian population (22), compounding the difficulty of data synthesis. Thirdly, other members of the microbial community, such as the mycobiome and virome, which may both be of relevance to IBS (45, 46), were not evaluated. Finally, sensitivity analysis based on adherence, IBS subtype, duration, and dose of treatment was not possible owing to the small number of trials and lack of quantitative synthesis.

In conclusion, a low FODMAP diet led to altered abundances of a limited number of taxa in patients with IBS, although most effects were inconsistent. Clear shifts were observed for *Bifidobacteria*, with a consistently lower abundance after a low FODMAP dietary intervention. Amid speculation on its safety, microbiome changes induced by 3–4 wk of FODMAP restriction are specific for *Bifidobacteria* and do not involve broad changes to microbial composition and function. This should allay concerns about the safety of a short-term low FODMAP diet with regards to the colonic microenvironment. Consistent methodology and reporting will be important for identifying the precise effects of short- and long-term low FODMAP dietary interventions on the colonic microbiome and for elucidating potential mechanisms of effect.

## Supplementary Material

nqac176_Supplemental_File

## Data Availability

Data described in the article, code book, and analytic code will be made available upon reasonable request to the corresponding author.

## Notes

The authors reported no funding received for this study.

Supplemental Tables 1–4 are available from the “Supplementary data” link in the online posting of the article and from the same link in the online table of contents at https://academic.oup.com/ajcn/.

Abbreviations used: BCFA, branched-chain fatty acid; FISH, fluorescence in situ hybridization; FODMAP, fermentable oligosaccharides, disaccharides, monosaccharides, and polyols; IBS, irritable bowel syndrome; MD, mean difference; rRNA, ribosomal RNA; SMD, standardized mean difference.
