# Supplementary material for: Effects of a low FODMAP diet on the colonic microbiome in irritable bowel syndrome: a systematic review with meta-analysis
Source: Am J Clin Nutr. 2022 Jun 21;116(4):943–52. doi: 10.1093/ajcn/nqac176 (PMC9535515; doi:10.1093/ajcn/nqac176)
Supplement: nqac176_Supplemental_File [file nqac176_supplemental_file.docx]

**ON-LINE SUPPLEMENTARY MATERIAL**

**Effects of a low FODMAP diet on the colonic microbiome in irritable bowel syndrome: a systematic review with meta-analysis**

Daniel So^1^, Amy Loughman^2^, Heidi M. Staudacher^2^

**Affiliations**

^1^ Department of Gastroenterology, Central Clinical School, Monash University and Alfred Health, Victoria, Australia

^2^ Food & Mood Centre, Institute for Mental and Physical Health and Clinical Translation (IMPACT), Deakin University, Victoria, Australia

**Supplementary Table 1**: Search algorithm: MEDLINE via OVID

| 1. exp Irritable Bowel Syndrome/ 2. Colonic Diseases, Functional/ 3. irritable bowel syndrome.tw,kf. 4. ibs.tw,kf. 5. functional gastrointestinal.tw,kf. 6. functional bowel.tw,kf. 7. irritable colon.tw,kf. 8. spastic colon.tw,kf. 9. 1 or 2 or 3 or 4 or 5 or 6 or 7 or 8 10. exp Diet, Gluten-Free/ 11. gluten free.tw,kf. 12. exp Prebiotics/ 13. prebiotic*.tw,kf. 14. exp Fructans/ 15. fructan*.tw,kf. 16. exp Dietary Fiber/ 17. dietary fibre*.tw,kf. 18. dietary fiber*.tw,kf. 19. exp Dietary Carbohydrates/ 20. carbohydrate*.tw,kf. 21. (carbohydrate adj2 polymer*).tw,kf. 22. ((non-starch or nonstarch) adj (poly-saccharide* or polysaccharide*)).tw,kf. 23. exp Diet, Carbohydrate-Restricted/ 24. fodmap*.tw,kf. 25. (fermentable adj2 carbohydrate*).tw,kf. 26. exp Oligosaccharides/ 27. oligosaccharide*.tw,kf. 28. exp Inulin/ 29. inulin.tw,kf. 30. exp Psyllium 31. psyllium.tw,kf. 32. ispagula.tw,kf. | 1. exp Sweetening Agents/ 2. sweetner*.tw,kf. 3. exp Sugar Alcohols/ 4. polyol*.tw,kf. 5. exp Fructose/ 6. fructose.tw,kf. 7. exp Lactose/ 8. lactose.tw,kf. 9. 10 or 11 or 12 or 13 or 14 or 15 or 16 or 17 or 18 or 19 or 20 or 21 or 22 or 23 or 24 or 25 or 26 or 27 or 28 or 29 or 30 or 31 or 32 or 33 or 34 or 35 or 36 or 37 or 38 or 39 or 40 10. exp Microbiota/ 11. exp Gastrointestinal Microbiome/ 12. microbio*.tw,kf. 13. gut flora.tw,kf. 14. exp Mycobiome/ 15. mycobio*.tw,kf. 16. virome.tw,kf. 17. exp Fatty Acids, Volatile/ 18. (short chain fatty acid*).tw,kf. OR (short-chain fatty acid*).tw,kf. 19. exp Butyrates/ 20. butyrate*.tw,kf. 21. 42 or 43 or 44 or 45 or 46 or 47 or 48 or 49 or 50 or 51 or 52 22. (faecal or fecal).tw,kf. 23. (bacteri* or flora).tw,kf. 24. 54 and 55 25. 53 or 56 26. 9 and 41 and 57 |
| --- | --- |

**Supplementary Table 2**: Search algorithm: EMBASE via Elsevier

| 1. 'irritable colon'/exp 2. 'intestine function disorder'/exp 3. "irritable bowel syndrome":ti,ab,kw 4. ibs:ti,ab,kw 5. “functional gastrointestinal”:ti,ab,kw 6. “functional bowel”:ti,ab,kw 7. “irritable colon”:ti,ab,kw 8. “spastic colon”:ti,ab,kw 9. #1 OR #2 OR #3 OR #4 OR #5 OR #6 OR #7 OR #8 10. 'gluten free diet'/exp 11. “gluten free”:ti,ab,kw 12. 'prebiotic agent'/exp 13. prebiotic*:ti,ab,kw 14. 'fructan'/exp 15. fructan*:ti,ab,kw 16. 'dietary fiber'/exp 17. “dietary fibre*”:ti,ab,kw 18. “dietary fiber*”:ti,ab,kw 19. 'carbohydrate'/exp 20. carbohydrate*:ti,ab,kw 21. carbohydrate NEAR/2 polymer* 22. ('non starch' OR nonstarch) NEAR/1 ('poly saccharide*' OR polysaccharide*) 23. 'low FODMAP diet'/exp 24. fodmap*:ti,ab,kw 25. fermentable NEAR/2 carbohydrate* 26. ‘oligosaccharide'/exp 27. oligosaccharide*:ti,ab,kw 28. 'inulin'/exp 29. inulin:ti,ab,kw 30. 'carbohydrate'/exp 31. Dietary NEAR/2 carbohydrate 32. 'ispagula'/exp 33. ispagula:ti,ab,kw 34. psyllium:ti,ab,kw 35. 'sweetening agent'/exp 36. sweetener*:ti,ab,kw 37. polyol*:ti,ab,kw 38. 'fructose'/exp | 1. fructose:ti,ab,kw 2. 'lactose'/exp 3. lactose:ti,ab,kw 4. #10 OR #11 OR #12 OR #13 OR #14 OR #15 OR #16 OR #17 OR #18 OR #19 OR #20 OR #21 OR #22 OR #23 OR #24 OR #25 OR #26 OR #27 OR #28 OR #29 OR #30 OR #31 OR #32 OR #33 OR #34 OR #35 OR #36 OR #37 OR #38 OR #39 OR #40 OR #41 5. 'colon flora'/exp 6. 'intestine flora'/exp 7. 'feces microflora'/exp 8. 'microbiome'/exp 9. microbio*:ti,ab,kw 10. 'mycobiome'/exp 11. myocbio*:ti,ab,kw 12. 'volatile fatty acid'/exp 13. "short chain fatty acid*":ti,ab,kw OR "short-chain fatty acid*":ti,ab,kw 14. 'butyric acid'/exp 15. butyrate*:ti,ab,kw 16. #43 OR #44 OR #45 OR #46 OR #47 OR #48 OR #49 OR #50 OR #51 OR #52 OR #53 17. faecal OR fecal:ti,ab,kw 18. bacteri* OR flora:ti,ab,kw 19. #55 AND #56 20. #54 OR #57 21. #9 AND #42 AND #57 22. random* OR factorial OR crossover OR placebo OR blind OR blinded OR assign OR assigned OR allocate OR allocated OR 'crossover procedure'/exp OR 'double-blind procedure'/exp OR 'randomized controlled trial'/exp OR 'single-blind procedure'/exp 23. #59 AND #60 24. #61 AND [embase]/lim |
| --- | --- |

**Supplementary Table 3**: Search algorithm: CENTRAL

| 1. MeSH descriptor: [Irritable Bowel Syndrome] explode all trees 2. MeSH descriptor: [Colonic Diseases, Functional] explode all trees 3. irritable bowel syndrome 4. ibs 5. functional gastrointestinal 6. functional bowel 7. irritable colon 8. spastic colon 9. #1 or #2 or #3 or #4 or #5 or #6 or #7 or #8 10. MeSH descriptor: [Diet, Gluten-Free] explode all trees 11. gluten free 12. MeSH descriptor: [Prebiotics] explode all trees 13. prebiotic* 14. MeSH descriptor: [Fructans] explode all trees 15. fructan* 16. MeSH descriptor: [Dietary Fiber] explode all trees 17. dietary fibre* 18. dietary fiber* 19. MeSH descriptor: [Dietary Carbohydrates] explode all trees 20. carbohydrate* 21. carbohydrate near/2 polymer* 22. ((non-starch or nonstarch) near (poly-saccharide* or polysaccharide*)) 23. MeSH descriptor: [Diet, Carbohydrate-Restricted] explode all trees 24. fodmap* 25. fermentable near/2 carbohydrate* 26. MeSH descriptor: [Oligosaccharides] explode all trees 27. oligosaccharide* 28. MeSH descriptor: [Inulin] explode all trees | 1. inulin 2. MeSH descriptor: [Psyllium] explode all trees 3. psyllium 4. ispaghula 5. MeSH descriptor: [Sweetening Agents] explode all trees 6. sweetener* 7. polyol* 8. MeSH descriptor: [Fructose] explode all trees 9. fructose 10. MeSH descriptor: [Lactose] explode all trees 11. lactose 12. #10 or #11 or #12 or #13 or #14 or #15 or #16 or #17 or #18 or #19 or #20 or #21 or #22 or #23 or #24 or #25 or #26 or #27 or #28 or #29 or #30 or #31 or #32 or #33 or #34 or #35 or #36 or #37 or #38 or #39 13. MeSH descriptor: [Microbiota] explode all trees 14. MeSH descriptor: [Gastrointestinal Microbiome] explode all trees 15. microbio* 16. gut flora 17. MeSH descriptor: [Mycobiome] explode all trees 18. mycobio* 19. virome 20. MeSH descriptor: [Fatty Acids, Volatile] explode all trees 21. short chain fatty acid* OR short-chain fatty acid* 22. MeSH descriptor: [Butyrates] explode all trees 23. butyrate* 24. #41 or #42 or #43 or #44 or #45 or #46 or #47 or #48 or #49 or #50 or #51 25. (faecal or fecal) 26. (bacteri* or flora) 27. #53 and #54 28. #52 and #55 29. #9 and #40 and #56 |
| --- | --- |

**Supplementary Table 4**: Search algorithm: Web of Science

| 1. TS=(“irritable bowel syndrome”) 2. TS=(“functional gastrointestinal”) 3. TS=(“functional bowel”) 4. TS=(“irritable colon”) 5. TS=(“spastic colon”) 6. #5 OR #4 OR #3 OR #2 OR #1 7. TS=(gluten Near/2 free) 8. TS=(prebiotic*) 9. TS=(fructan*) 10. TS=("dietary fibre*") 11. TS=("dietary fiber*") 12. TS=("dietary carbohydrate*") 13. TS=(carbohydrate Near/2 polymer*) 14. TS=((non-starch OR nonstarch) near/2 (poly-saccharide* OR polysaccharide)) 15. TS=(carbohydrate* Near/2 restrict*) 16. TS=(fodmap*) 17. TS=(fermentable Near/2 carbohydrate*) 18. TS=(oligosaccharide*) | 1. TS=(inulin) 2. TS=(psyllium) 3. TS=(ispaghula) 4. TS=(sweetener*) 5. TS=(“sweetening agent*”) 6. TS=(polyol*) 7. TS=(fructose) 8. TS=(lactose*) 9. #26 OR #25 OR #24 OR #23 OR #22 OR #21 OR #20 OR #19 OR #18 OR #17 OR #16 OR #15 OR #14 OR #13 OR #12 OR #11 OR #10 OR #9 OR #8 OR #7TS=(microbio*) 10. TS=(“gut flora”) 11. TS=(mycobio*) 12. TS=(virome) 13. TS=(“short chain fatty acid*” OR “short-chain fatty acid*”) 14. TS=(butyrate*) 15. #33 OR #32 OR #31 OR #30 OR #29 OR #28 16. TS=((faecal OR fecal) AND (bacteri* OR flora)) 17. #35 OR #34 18. #36 AND #27 AND #6 |
| --- | --- |
